# Crowding Changes Appearance

**DOI:** 10.1016/j.cub.2010.01.023

**Published:** 2010-03-23

**Authors:** John A. Greenwood, Peter J. Bex, Steven C. Dakin

**Affiliations:** 1 UCL Institute of Ophthalmology, University College London, 11-43 Bath Street, London EC1V 9EL, UK; 2 Schepens Eye Research Institute, Harvard Medical School, Boston, MA 02114, USA

**Keywords:** SYSNEURO

## Abstract

Crowding is the breakdown in object recognition that occurs in cluttered visual environments [1–4] and the fundamental limit on peripheral vision, affecting identification within many visual modalities [5–9] and across large spatial regions [10]. Though frequently characterized as a disruptive process through which object representations are suppressed [11, 12] or lost altogether [13–15], we demonstrate that crowding systematically changes the appearance of objects. In particular, target patches of visual noise that are surrounded (“crowded”) by oriented Gabor flankers become perceptually oriented, matching the flankers. This was established with a change-detection paradigm: under crowded conditions, target changes from noise to Gabor went unnoticed when the Gabor orientation matched the flankers (and the illusory target percept), despite being easily detected when they differed. Rotation of the flankers (leaving target noise unaltered) also induced illusory target rotations. Blank targets led to similar results, demonstrating that crowding can induce apparent structure where none exists. Finally, adaptation to these stimuli induced a tilt aftereffect at the target location, consistent with signals from the flankers “spreading” across space. These results confirm predictions from change-based models of crowding, such as averaging [16], and establish crowding as a regularization process that simplifies the peripheral field by promoting consistent appearance among adjacent objects.

## Graphical abstract

**Graphical Abstract:**

**Figure:**
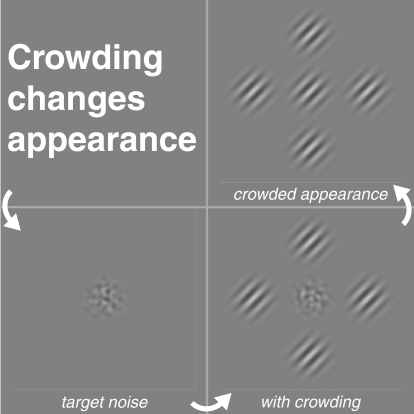

**Highlights:**

► Patches of visual noise become perceptually oriented when crowded by Gabor elements ► Changes from crowded-noise targets to Gabors go unnoticed when perceptually matched ► Adaptation to crowded change gives tilt aftereffects in the target location ► Crowding promotes consistency by regularizing the peripheral visual field

## Results

Although the conditions required for crowding are well established—including peripheral viewing [17, 18], close target-flanker proximity [2, 10, 17], and high target-flanker similarity [5, 19]—exactly how crowding occurs is unclear. A distinction can be drawn between models of crowding that rely on information loss, with crowded items either suppressed [11, 12] or lost [13–15], and change-based models such as averaging [16] and flanker substitution [20–22]. The former predict that crowding should have purely random effects; the latter predict a systematic interaction between target and flanker elements. Change-based models are therefore better able to explain the correlation between target identification errors and the structure of flanking elements [9, 16, 22–25]. However, these systematic effects could reflect behavioral strategies that, for instance, lead observers to report the average of a stimulus array under conditions of high uncertainty or to simply report the flankers because of information loss at the target location. Clear demonstration of a genuine change in the appearance of crowded targets has yet to be made.

**Figure 1:**
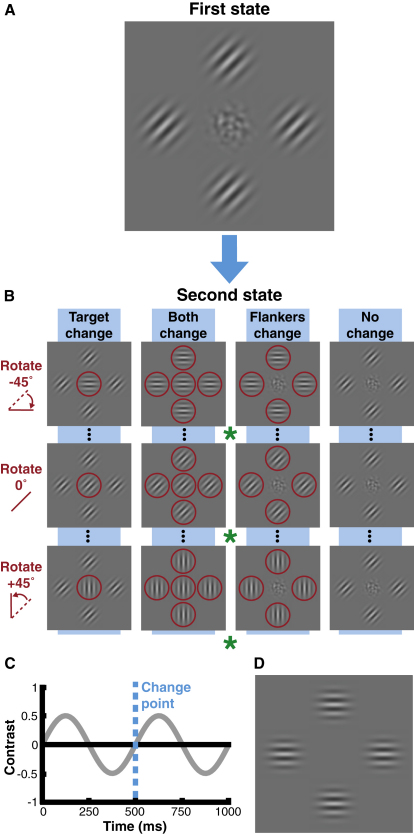
Stimuli and Procedure for the Change-Detection Experiments (A) Experiments began with the target noise surrounded by four identical flankers at one of four orientations (45° depicted). Closing one eye and viewing the stimulus peripherally—by maintaining fixation on one of the green asterisks (depending on viewing distance)—allows one to see the effect of crowding: the target noise should become perceptually oriented. (B) In one quarter of all trials, noise stimuli persisted throughout (no change). The remaining conditions involved a stimulus change (red circles). For the target-change condition, a Gabor was introduced at the target location, with an orientation between ±45° relative to the flankers. In the both-change condition, flankers rotated to match the introduced target. The flankers-change condition involved the rotation of flankers without a change in the target noise. All conditions were interleaved, and observers simply indicated whether the target had changed. (C) Time course of a single trial. Stimuli counterphase flickered, with changes occurring midway through the trial when stimuli were at mean luminance. (D) The initial stimulus configuration with a blank target (0° base depicted). Change conditions were as in (B), substituting blank regions for the noise patches.

Because flankers necessarily drive systematic effects, the clearest expression of target change is likely to occur when the target is noisy or even absent. To examine this, we constructed target patches of isotropic bandpass-filtered noise flanked by oriented Gabor stimuli (Figure 1A; see Experimental Procedures). We report that crowding induces target noise patches to appear oriented, matching the appearance of flankers to an extent that is indistinguishable from physically oriented stimuli. This can be seen in Figure 1A (see also Movie S1 available online). The appearance of the target noise patch is apparent when fixated directly, but peripheral viewing of the stimulus (by fixating one of the green asterisks monocularly) should make the target appear oriented—the stimulus may now appear to be composed of five oriented patches, or the target may blend with the flankers to form a single oriented texture.

**Figure 2:**
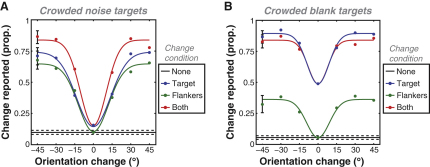
Crowded Change Detection Data show the proportion of trials for which change was reported (pooled across three observers). Error bars indicate 95% confidence intervals. (A) Change detection with target noise. Data are plotted as a function of the orientation change introduced, relative to the base orientation of the flankers. No-change trials gave a small proportion of false alarms (solid black line). In target-change (blue points) and both-change (red points) conditions, change detection was poor (approaching false alarm rates) when introduced target Gabors matched the initial flanker orientation (0° change). Detection improves with increasing orientation difference between the introduced Gabor and the flanker orientation (i.e., the perceived orientation of the crowded noise). Large rotation of the flankers in the flankers-change condition (green points) also led subjects to report target change, consistent with the flankers inducing an illusory rotation of the target noise. (B) Change detection with a blank target, plotted as in (A). The pattern of results is similar to that observed with crowded noise (i.e., introduced Gabors were most often missed when they matched the initial flanker orientation), albeit with a lower magnitude (meaning both fewer errors overall and a lower frequency of illusory rotations).

To examine observers' perceptual experience of these stimuli, we utilized a change-detection paradigm. Because observers can detect changes in crowded targets despite being impaired in their identification [26], change detection offers an indirect but effective measure of the percept of crowded stimuli without requiring subjective judgments (which are difficult to specify and/or quantify). Observers reported when a crowded noise patch was swapped for an oriented Gabor, either with or without concurrent changes in the flankers (Figure 1B). Because temporal transients can signal change [27, 28], stimulus contrast counterphased, with all changes taking place when stimuli passed through zero contrast (Figure 1C). On an equal proportion of trials, the noise target either persisted (no-change or flankers-change conditions; Movies S1 and S2) or was swapped midway for an oriented Gabor (target-change and both-change conditions; Movies S3–S5). False alarms (the frequency of change reported when there was none) were determined from the no-change condition and were below 10% for both uncrowded (Figure S1) and crowded noise patches (Figure 2A; black line). Under crowded conditions, changes between noise targets and Gabors (target change; blue points in Figure 2A) were rarely detected when the substituted Gabor matched the flanker orientation (with performance approaching the false alarm rate). We postulate that this is a consequence of the perceptual similarity between substituted Gabors and the illusory orientation of the crowded noise. Accordingly, changes were easily detected when substituted Gabors differed from the perceived target orientation.

This explanation assumes that observers compared their percepts from the two stages of each trial. However, these results could also arise from flankers inhibiting the Gabors introduced in the second stage of target-change trials [11, 12]—because crowding is orientation tuned [5], introduced Gabors could be inhibited based on their similarity to the flankers. A reduction in the visibility of these Gabors through masking [17, 29–31] might also produce these results. To control for this possibility, we included trials in which the flankers rotated in the second stage to match the introduced Gabors (both change; Figure 1B). If performance in the target-change condition arose through inhibition of the introduced Gabors, performance in the both-change condition should be uniformly poor as a result of this target-flanker match. However, as shown in Figure 2A (red points), the pattern of data from the two conditions is identical, consistent with observers comparing the introduced Gabors and their percept of the crowded target. We also tested the spatial extent of these effects by varying target-flanker separation and report interference zones spanning 6° (Figure S2A). In line with prior estimates of crowding [2, 10, 17], this is equivalent to 0.4 × the target eccentricity. Additionally, our stimulus configuration produces a minimal effect on detection thresholds (Figure S2B), contrary to the predictions of both masking and simple inhibition.

If, as we hypothesize, crowding induces the target noise to appear oriented, observers should also perceive an illusory rotation when the flankers rotate without a physical change in the target noise (flankers change; Figure 1B). This is indeed the case (green points; Figure 2A), with the highest rate of reported change occurring with large flanker rotations. The similarity between these data and observers' detection of introduced Gabors in other conditions suggests that these illusory changes are indistinguishable from physical changes. Our results are therefore mutually consistent with the notion that crowding alters appearance: here, isotropic stimuli assume an illusory orientation similar to the flankers'.

Because flanking elements are likely to drive this change in appearance, it might be possible to induce similar effects without a target. To test this, we repeated the procedure with a blank target instead of noise (Figure 1D). Viewed peripherally, this arrangement can give the faint appearance of oriented structure in the target region. For the change-detection paradigm, the target remained continuously blank in the no-change and flankers-change conditions and was swapped for a Gabor in the remaining conditions (as in Figure 1B). Results are plotted in Figure 2B and show a similar pattern to the crowded-noise experiment, albeit at a lower magnitude. As before, in target-change and both-change conditions, changes were most often missed when the substituted Gabor matched the flankers' orientation but were easily detected when substituted Gabors were dissimilar. However, both functions are now shifted upwards because of the lower likelihood of missed changes, consistent with the perceived orientation being more weakly induced in the target region. Similarly, the downward shift in the flankers-change data demonstrates a reduced rate of illusory changes, though the overall pattern was similar (with changes reported most often for large rotations). These results demonstrate that although target noise facilitates the expression of flanker-induced changes in appearance—perhaps similar to the way dynamic test patterns can reveal motion aftereffects [32]—a target is not required for crowding to occur.

**Figure 3:**
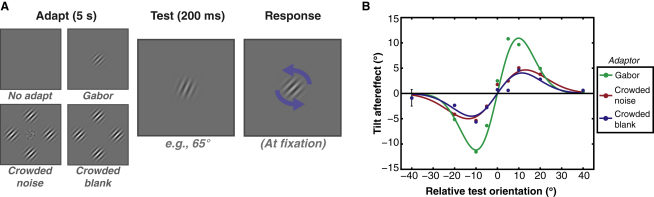
Adaptation to Crowded Stimuli (A) Observers either remained unadapted or adapted to either a single 45° Gabor at the target location, a noise patch crowded by 45° flankers, or the flankers in isolation (“crowded blank”). Following 5 s adaptation, a Gabor was presented at the target location for 200 ms at one of several possible orientations. Observers rotated a response Gabor (at fixation) to match their percept of the test. (B) Changes in perceived orientation after adaptation, averaged across three observers. Negative values indicate clockwise rotations and positive values indicate counterclockwise rotations; error bars depict ±1 standard error of the mean. Adaptation to a single Gabor (green points) produced repulsion in perceived orientation that peaks at test orientations ±10°–15° from the adaptor. Adaptation to either crowded noise (red points) or crowded blanks (blue points) produces the same pattern at a lower magnitude, consistent with the presence of an oriented signal at the target location.

Our results indicate that crowding can induce an orientation-selective change in the representation of the target. If this process engages the same low-level mechanisms that signal physical orientation, then prolonged viewing of our stimuli should induce adaptation. Ordinarily, adaptation to an oriented target produces a tilt aftereffect (TAE; [33]): the perceived orientation of subsequently viewed test stimuli is repulsed away from the adaptor. We examined whether crowding-induced changes in appearance could induce a TAE by having observers adapt to either (1) an uncrowded target Gabor, (2) crowded noise, or (3) a crowded blank region, followed by a single test Gabor on each trial (Figure 3A). Subjects indicated the apparent orientation of the peripheral test pattern by rotating a Gabor (at fixation) to match their percept. Postadaptation responses were then subtracted from preadaptation responses to measure the TAE.

As shown in Figure 3B, adaptation to an isolated target Gabor produced a robust TAE, with a maximum repulsion of ±10° at test orientations differing by ±10°–15° from the adaptor (consistent with prior studies of the peripheral TAE [34]). Following adaptation to either crowded-noise or crowded-blank regions, the same pattern was evident, peaking with a lower magnitude of ±5°. Here, the perceived orientation of the test was repelled from the orientation of the adapting flankers rather than from any physical structure at the target location. This effect of adaptation was not restricted to perceived orientation, with some elevation of contrast-detection thresholds also evident (Figure S3). Concurrent eye tracking further demonstrated that eye movements during adaptation (which might have shifted flankers into the target vicinity) cannot explain these results (Figure S4). Rather, these aftereffects are consistent with earlier findings that the spatial spread of adaptation becomes increasingly broad as adapting stimuli move further into the periphery [35]. We suggest that this spread in orientation signals contributes to crowding. Accordingly, manipulations that do not produce crowding (e.g., a target Gabor with orthogonal flankers [5]) produce a TAE that is indistinguishable from that induced with a similar target in isolation (Figure S5).

## Discussion

Our results demonstrate that crowding produces a change in object appearance: when crowded by Gabors, patches of isotropic noise assume the orientation of the flankers. Using a change-detection paradigm, we report that Gabors introduced at the target location go largely unnoticed when their orientations match this illusory percept but are easily detected when they differ from it (Figure 2A). Rotation of the flankers also caused an illusory rotation of the target noise, consistent with a crowding-induced orientation that is indistinguishable from physically oriented stimuli. Similar effects were apparent with blank targets (Figure 2B), providing the first demonstration of crowding without a target. Finally, adaptation to both crowded-noise and crowded-blank regions produced a tilt aftereffect (Figure 3B), consistent with the flankers' orientation being introduced at the target location. Together, these results suggest that crowding is a process that actively promotes perceptual similarity between adjacent regions of the visual field.

These findings are inconsistent with several current explanations of crowding. First, models that rely on information loss through insufficient resolution of the attentional spotlight [13–15] predict little to no systematic target changes, contrasting with the strong percept elicited by our simple stimuli. Our results are also inconsistent with models in which target and flanker locations are lost through processes such as a misdirected attentional spotlight [23, 36]. Errors in positioning attention should be either constant (if localization errors are stimulus independent) or reduced in the presence of target noise (compared with blank targets, because the target noise would provide additional positional information). Gross spatial uncertainty thus incorrectly predicts either less crowding with noise targets or no effect of target identity at all. The robust TAE observed after crowded adaptation is also inconsistent with target-flanker mislocalizations, because attentional allocation should not affect retinotopic adaptation processes. In short, our results argue strongly against crowding models based solely on information loss.

Our results are also inconsistent with inhibition-based models of crowding [11, 12] and explanations based on masking [17, 29–31]. If the flankers suppressed the target, performance should have been uniformly poor in the both-change condition (Figure 1B) where the flankers rotated to match the introduced Gabor. That performance was identical to the target-change condition (Figure 2A) indicates that observers performed the task by comparing their percept of the crowded stimulus and the introduced Gabor. As an alternative, one could argue that the flankers inhibit dissimilar orientations to promote similarity in the target location. Although this could produce our results by creating an imbalance in the population response to the target noise, dissimilarity-based inhibition is inconsistent with the known selectivity of crowding. That is, stronger crowding is observed with increased target-flanker similarity and not vice versa [5, 19]. We can thus exclude inhibition as the primary mechanism of crowding. These results are similarly inconsistent with reductions in stimulus visibility related to masking, in conjunction with the broad spatial extent of our change-detection effects and the minimal effect on detection thresholds (Figure S2). Nonetheless, there is clearly some effect of clutter on stimulus visibility when flankers closely abut the target (Figure S2B; [17, 37]). These masking effects (on stimulus detection) may interact with crowding effects (on identification) at the closest target-flanker separations. An increase in the strength of masking could therefore cause crowded changes in target appearance to be reduced or even eliminated, though this was not the case with our stimuli.

The changes we observed in crowded target appearance are consistent with the correlation between target identification errors and the identity of flanking elements [9, 16, 22–25], suggesting that these systematic effects reflect a genuine change in the target representation rather than behavioral strategies aimed at overcoming uncertainty. These changes follow predictions from two change-based models of crowding. The first is flanker substitution, where either flanker features [11, 20] or flankers in their entirety [21, 22] replace the target. The second is a compulsory average of target and flanker signals [16]. Both models require that flanker identities propagate into the target location but differ in the way that target and flanker signals interact. Substitution predicts that flankers overwrite the target and could thus predict both changes in appearance and orientation-selective adaptation. An average of target and flanker identities could similarly mimic our results, because averaging the flankers with noise (arising from either the visual system or the stimulus) would also replicate the flanker identity. Recent experiments demonstrate that a weighted average of noisily-encoded target and flanker feature positions can account for both the threshold elevation and the flanker-directed biases in judgments of the feature positions within letter-like elements, whereas flanker substitution predicts erroneously extreme feature positions [9]. We therefore favor an explanation where target flanker averaging produces both systematic and random aspects of crowding as a result of the inherent featural uncertainty of the periphery.

Several cortical mechanisms could subserve these effects. The first is propagation via lateral interactions within primary visual cortex [38]. Although this may appear to conflict with the minimal effects on the adaptive strength [13, 39] and contrast-detection thresholds of crowded targets [17, 29], our results suggest a potential reinterpretation of these results: crowding could produce a change in the identity of crowded targets without affecting their perceived contrast. However, the extent of horizontal connections scale to only 0.1–0.2 × the target eccentricity [3], rather than the requisite ∼0.4–0.5 × scaling seen here and elsewhere [2, 17]. A second possibility is that target changes occur through pooling within large receptive fields, likely within cortical areas such as V4 [40], though multiple regions may be involved through both feedforward and feedback connectivity [41]. We consider this to be the best current explanation of our findings.

Changes in the appearance of crowded targets bear a strong resemblance to the filling-in that occurs when regions of texture perceptually complete across either homogeneous target regions or the blind spot [42, 43]. Filling-in shares many characteristics with crowding, including an increased magnitude in the periphery, orientation tuning, binocular mechanisms, and occurrence across the blind spot (filling-in [42–45]; crowding [5, 19, 46]). Although the timescale of filling-in may be longer than crowding [45], it is likely that these processes are related. Changes in crowded target appearance may also underlie many effects in the change-detection literature [28].

Finally, the change-based processes observed herein demonstrate that crowding may not serve a purely disruptive role in visual perception. Rather than adding noise or suppressing target elements, crowding appears to explicitly promote perceptual similarity between adjacent regions of the peripheral visual field. This could involve the representation of large spatial regions as if they were texture (essentially preparing a statistical description [47, 48]), a process that could allow a more efficient representation of information given the low spatial sampling and high featural uncertainty of the periphery.

## Experimental Procedures

### Observers

Three experienced observers participated in the experiments: two of the authors (J.A.G. and S.C.D.) and one naive observer. All had normal or corrected-to-normal visual acuity.

### Apparatus

Experiments were programmed with MATLAB (MathWorks) on a Macintosh computer running PsychToolbox [49]. Stimuli were presented on a cathode ray tube monitor (LaCie Electron Blue 22) with a resolution of 1152 × 870 pixels and a refresh rate of 75 Hz, fitted with a Bits++ box (Cambridge Research Systems) to give 14-bit contrast resolution. The monitor was calibrated with a Minolta LS110 photometer and linearized with look-up tables to give a mean and maximum luminance of 50 and 100 cd/m^2^, respectively. Stimuli were viewed monocularly with the dominant eye from a distance of 57 cm. Experiments took place in a dark room, with responses made with either the keyboard (change detection) or mouse (adaptation).

### Stimuli and Procedures

In all experiments, stimulus elements were either Gabors or patches of filtered noise (as in Figure 1A). Target noise stimuli were constructed from white noise that was convolved with a log Gaussian filter in the spatial frequency domain. This filtering was isotropic for orientation, with a peak spatial frequency of 2.5 cycles/degree (c/deg) and a bandwidth (s) of 1 octave. Gabor stimuli were also presented with a spatial frequency of 2.5 c/deg. The Gaussian window around both Gabors and noise elements had a standard deviation of 0.4°, and elements were presented at 50% Michelson contrast. All elements were counterphase flickered at 2 Hz with the same temporal phase (see Figure 1B).

Targets were presented 15° in the upper visual field. Under crowded conditions, four flankers were positioned above, below, to the right, and to the left of the target. Targets and flankers had a center-to-center separation of 2.75°, which, at 15° eccentricity, falls well within the region of interference [10, 17]. When targets were absent, the central noise patch was left blank at the mean luminance. Identical configurations were used for both change-detection and adaptation paradigms.

In the change-detection experiments, each 1 s trial was notionally divided into two 500 ms stages (Figure 1C). With target noise, the first stage contained the target noise patch surrounded by four Gabors (Figure 1A). Flanker orientations were always matched and were set initially to be either 0° (horizontal), 45° (tilted to the right), 90°, or 135°. After 500 ms, the counterphase time course of all elements reached mean luminance, and one of four conditions were initiated (Figure 1B). In the no-change condition, both target noise and flanking Gabors remained unchanged. For the target-change condition, the target noise was swapped for a Gabor element with an orientation that differed from the flankers by ±45° in 15° steps. The flankers-change condition left the central noise unchanged, with flankers rotated en masse by between ±45° relative to their initial orientation. Finally, the both-change condition involved both the appearance of a target Gabor and the rotation of the flanking Gabors so that the entire ensemble shared the same orientation. To examine the influence of isolated flankers, we also ran experiments with a blank target region (Figure 1D). Such trials were otherwise identical to those of the main experiment, incorporating the four change conditions (replacing target noise with a blank region where appropriate). In both cases, each orientation difference was presented 20 times for each change condition (five times at each of four base orientations), interleaved randomly to make 560 trials per block. Observers completed three blocks for each experiment, with crowded-noise and crowded-blank stimuli tested separately. Data were recorded as the proportion of trials in which change was reported and were pooled across observers (because all showed a similar pattern). Each data set was fit with an inverted Gaussian profile, and 95% confidence intervals were determined via a bootstrapping procedure with 1000 repetitions [50].

Stimuli were again at 15° eccentricity for the adaptation experiments, with similar parameters. Adaptation stimuli were either a single 45° Gabor at the target location, target noise surrounded by four flankers (each oriented at 45°), or the isolated flankers with a blank target region. Flankers were presented at 100% Michelson contrast to maximize crowding [19]. These stimuli counterphase flickered at 2 Hz for 5 s per trial, followed by a test interval for 200 ms. Test intervals contained a single Gabor at the target location with an orientation between 5° and 85°. A response Gabor then appeared at fixation, and observers adjusted its orientation with the mouse until it matched their percept of the peripheral test stimulus. This adapt-test cycle then continued, with the first 10 trials discarded to enable the buildup of adaptation [13, 39]. Each test orientation was presented 20 times per block to give 190 trials, including practice. Observers repeated each adaptation condition three times in random order, with breaks taken when switching between conditions. For each test orientation, the mean perceived orientation was calculated. Data were again pooled across observers and fit with the first derivative of a Gaussian, given the absence of attractive effects in the peripheral TAE [34].

## Supplemental Information

Document S1. Supplemental Experimental Procedures and Five Figures

Movie S1. An Example Crowded-Noise Stimulus and No-Change Trial The stimulus contains a target patch of filtered noise surrounded by four identical flankers at one of four orientations (45° depicted). Viewing the stimulus peripherally should allow one to see the effect of crowding: the target noise should become perceptually oriented. The effect is strongest with monocular viewing.

Movie S2. An Example Flankers-Change Trial, with a Flanker Rotation of +45° The base orientation of the flankers is rotated after 500 ms, leaving the noise unchanged.

Movie S3. An Example Target-Change Trial with a Target Gabor That Is Identical to the Flankers (0° Orientation Change) After 500 ms, the target noise patch is swapped for an oriented Gabor.

Movie S4. An Example Target-Change Trial with a Target Gabor Rotated +45° from the Base Orientation of the Flankers As in other conditions, the change point occurs 500 ms into the trial.

Movie S5. An Example Both-Change Trial (+45° Orientation Change) Here, as well as the introduction of a target Gabor, the flankers rotate to match the introduced orientation.
