# Supplementary material for: Crowding Changes Appearance
Source: Curr Biol. 2010 Mar 23;20(6):496–501. doi: 10.1016/j.cub.2010.01.023 (PMC2849014; doi:10.1016/j.cub.2010.01.023)
Supplement: Document S1. Supplemental Experimental Procedures and Five Figures [file mmc1.pdf]

Current Biology, Volume *20*

Supplemental Information

Crowding Changes Appearance

John A. Greenwood, Peter J. Bex, and Steven C. Dakin

Supplemental Experimental Procedures

Change-Detection Experiments

Uncrowded Change Detection

The primary finding of this study is that patches of non-oriented visual noise appear oriented when crowded by Gabor flankers. This was demonstrated through the failure of observers to report trials in which crowded noise patches were changed into Gabors with an orientation that matched the flankers. In this section we present data from an uncrowded change-detection experiment that rule out performance anisotropies as an alternative explanation for this finding. Stimuli and procedures were similar to those of the main experiment, except that only a single target patch was present (Supplementary Fig. 1A). Each 1 s trial began with an isolated noise patch present for 500 ms, after which one of two conditions could be initiated. In the *no change* condition, the target noise remained present for the remainder of the 1 s trial. In the *target change* condition, a Gabor was introduced at the target location with a given orientation. Unlike the crowded conditions of the main experiment, orientations here are defined absolutely (0-179°), rather than relative to the flanker orientation. Each condition was presented 144 times in a given block; with each orientation in the *target change* condition presented 12 times. The three observers repeated each block three times.

As before, data were the proportion of trials in which change was reported. As all three observers showed an identical pattern of performance, data were pooled before a wrapped Gaussian profile was fitted and 95% confidence intervals determined using a 1000-repetition bootstrap [1]. The false alarm rate was determined from the *no change* condition, and was again less than 10% (Supplementary Fig. 1B; black line). In the *target change* condition, between 89-99% of changes were correctly reported, regardless of which target orientation was introduced (Supplementary Fig. 1B; blue points). This isotropic, near-ceiling performance stands in contrast to the pattern obtained when target-noise patches were crowded with oriented Gabors, as in the main text.

Crowding-Induced Change and Masking

Although it is often difficult to distinguish crowding from similar processes such as masking [2, 3], several lines of evidence support the notion that our change-detection effects originate from crowding. First, consider the effect of masking on the target-noise patches in the first half of each trial (Fig. 1A). Because masking reduces sensitivity to objects of similar spatial

structure [2, 3], it should reduce the effective contrast of similarly-oriented structure in the isotropic noise which, if it were to have a perceptual consequence, would lead the noise to appear oriented *orthogonally* to the flankers. In contrast, the actual appearance of crowded-noise patches was that their orientation matched the flankers (see data in Fig. 2A and the appearance of Fig. 1A in the periphery). Second, consider the effect of masking on the oriented Gabors introduced in the second half of *target change* and *both change* trials (Fig. 1B). In the *target change* condition, it is possible that the flankers could have masked the Gabors introduced to the target location, reducing the visibility of similarly oriented targets and thus producing the pattern of data in Fig. 2A. However, as outlined in the main text, this is not true for the *both change* condition. If impaired performance in the *target change* condition resulted from inhibition of the introduced Gabors, performance in the *both change* condition should be uniformly poor, as the flankers were always rotated to match the introduced Gabors. As shown in Fig. 2A (red points), this is not the case: the pattern of data was identical to that of the *target change* condition, consistent with observers comparing the introduced Gabors and their percept of the crowded noise.

In order to test for the involvement of masking more directly, we first observe a critical signature of crowding in our change-detection task. Namely, the distance over which target and flankers interact is equal to approximately half the target eccentricity [2, 4]. By contrast, the spatial extent of masking scales with target size, regardless of eccentricity, such that masks need to be either overlapping or in close proximity to the targets [2, 5, 6]. It follows that if our change-detection results are a consequence of crowding, these effects should be evident across a wide range of target-flanker separations. The magnitude of effects with crowded noise and blank targets should also decrease at the same rate with increasing target-flanker separation. To assess this, we re-ran the *no change* and *target change* conditions (using both noise and blank targets) as a function of target-flanker separation. Gabors in the *target change* condition were only introduced at the same orientation as the flankers, as this produces the strongest effect. Target-flanker separations between 2.75 and 11 deg. were tested in steps of 1.375 deg, both with crowded-noise and crowded-blank stimuli. Each target-flanker separation was presented 20 times for each change condition (five times at each of the four base orientations), interleaved randomly to make 560 trials per block. Observers completed three blocks, with crowded-noise and crowded-blank stimuli tested separately.

For each target-flanker separation, the proportion of change reported is plotted in Supplementary Fig. 2a for both crowded-noise (dark blue) and crowded-blank (light blue) stimuli. For both target types, increasing target-flanker separation produced a modest decrease in the rate of false alarms in the *no change* condition (dashed lines). In the *target change* condition, detection of change is poorest at small target-flanker separations, and improves markedly with increasing separation. The magnitude of this effect is approximately halved in the absence of a target, but both effects show an identical spatial extent, reaching a half-width at half-height of 5.7 deg. with blank targets and 5.8 deg. for crowded noise (shown with grey lines). This gives *interference zones* (the region around a target where the presence of flankers interferes with target recognition) proportional to 0.4× the target eccentricity (of 15 deg.), consistent with our effects arising from crowding [2, 4].

We next sought to examine whether our target-flanker configuration produced *masking*, i.e. whether there was a decrease in stimulus visibility, and if so, whether the spatial extent of this effect was similar to that produced by crowded change detection. Prior studies suggest that while crowding impairs object identification across large spatial extents (scaled with eccentricity), the same stimuli have a much smaller effect on contrast-detection thresholds

that occurs only when flankers are close to the target [2, 3]. We thus examined the effect of our target-flanker configuration on the detection of an oriented Gabor in the target location, using a paradigm that resembled the change-detection experiments as closely as possible. Each trial in this detection paradigm consisted of two 500 ms intervals (equivalent to the two halves of change-detection trials), separated by 500 ms. One interval contained a target Gabor at a given luminance contrast; the other remained blank. Each interval was delineated with a beep and an inversion of the contrast polarity of the fixation cross, with observers required to indicate which interval contained the target. Crowded detection thresholds were assessed with the addition of four vertically oriented flankers at 50% contrast, present in both intervals of the task. Target-flanker separations between 2.75 and 8.25 deg. were tested in steps of 1.375 deg. Thresholds were assessed by varying the Michelson contrast of the target using a QUEST staircase [7] that converged on 82% correct identification with a maximum of 45 trials per condition. Staircases for uncrowded detection and crowded detection at each target-flanker separation were interleaved in each run. This procedure was repeated three times for each of the three observers.

All observers showed the same pattern of performance and data were thus pooled, as presented in Supplementary Fig. 2b. When uncrowded, detection thresholds were around 4.5% Michelson contrast (solid black line). Under crowded conditions (green points), thresholds rose to around 18% at the closest target-flanker separation (4× uncrowded threshold). Importantly, this degree of masking is insufficient to account for our change-detection results: a four-fold elevation of thresholds would be unlikely to mask the visibility of either the target-noise or the target-Gabors introduced in the *target change* and *both change* conditions, which were presented at 50% Michelson contrast (i.e. 11× detection threshold). Furthermore, this masking rapidly declines with increasing target-flanker separation – when fit with a cumulative Gaussian function, the half-width at half-height occurs at a 3.8 deg. separation. This corresponds to 0.25× the target eccentricity, a modest spread that is only slightly broader than that of prior investigations [2, 3]. The slight increase in spatial extent over these prior studies is likely to reflect our use of four flankers, compared with two, which has been shown to increase the strength of masking [8]. Nonetheless, the effect on contrast-detection thresholds drops off considerably more rapidly than the effect of crowding on change detection.

A potential issue with the above analysis is the different nature of the data being compared in the two experiments. Differences in the range and magnitude of these effects could differentially affect the estimation of their spatial extent. To give each data set an equal footing, sensitivity measures were thus calculated for each experiment prior to normalization. For the change-detection data, a single measure of sensitivity (d') can be derived for each target type by subtracting z scores for the proportion of change in the *no change* condition (false alarms) from z scores for the *target change* condition (hits). For the masking paradigm, sensitivity can be determined from the inverse of the threshold elevation ratios obtained initially by dividing crowded thresholds by this uncrowded baseline. Both datasets were then normalized by subtracting the minimum and dividing by the maximum. The resulting values, ranging from 0-1 and fit with cumulative Gaussian functions, are plotted in Supplementary Fig. 2c. While this does alter the spatial spread of these effects slightly, with masking now reaching a half-width at half-height at 4.6 deg., crowded-blank stimuli at 6 deg. and crowded-noise at 6.6 deg, the relative spatial extent of these effects remains the same – masking still drops off at a faster rate than either of the change-detection effects.

Although the flankers clearly produce some degree of masking, these results demonstrate that masking cannot account for the entirety of the change-detection effects reported herein. First, a reduction in the visibility of structure oriented similarly to the flankers cannot explain either the subjective appearance of crowded-noise stimuli or data from the *both change* condition. Second, the magnitude of masking that we observe is modest and would have been insufficient to completely reduce the visibility of our target stimuli. Finally, masking shows a considerably smaller spatial extent than crowded change detection. This distinction fits with the more general dichotomy between crowding and masking – the former affects identification, while the latter affects detection [2, 3]. Given that our change-detection task requires observers to compare their percept of the two intervals, it clearly falls within the realm of target identification and thus suffers from crowding. Nonetheless, it is worth noting that this distinction is not absolute – configurations consistent with 'crowding', as in the present study, can nonetheless produce significant levels of masking. It is clear however that the effects of masking cannot completely encapsulate the effects of crowded change-detection.

Adaptation Experiments

Contrast Detection Thresholds

As outlined in the main text, our results suggest that crowding can produce an orientation-selective change in the representation of target stimuli. Consistent with this view, adaptation to both crowded-noise and crowded-blank stimuli produced a robust tilt aftereffect at the target location (Fig. 3). However, while these results are consistent with a spreading of the flanker signals, the latter result differs from previous studies that have examined the effect of crowded adaptors on contrast-detection thresholds. Specifically, adaptation to crowded Gabor targets has been shown to produce threshold elevation for the detection of similarly oriented Gabors that is either identical [9] or reduced in magnitude [10] compared with adaptation to uncrowded Gabors. Crowding in this case appears to have either no effect, or to operate as a suppression of the adapting signal, unlike the additive effect produced by the flankers in our experiments.

One reason for this discrepancy could relate to the more general debate regarding the locus of crowding and its effects on detection and identification. As above, although crowding impairs object identification, it has less impact on contrast-detection thresholds for the same objects [2, 3]. Were this to carry across to adaptation, crowded adaptors could affect post-adaptation identification with little-to-no effect on detection. Our use of the tilt aftereffect (measured via perceived orientation) might thus have biased our results towards these identification mechanisms, compared with prior work examining effects on post-adaptation detection thresholds. In order to test this hypothesis, we also examined the effect of adaptation to crowded-noise and crowded-blank stimuli on contrast-detection thresholds for oriented test stimuli in the target location.

As in the adaptation experiment in-text (Fig. 3), adaptation stimuli were presented counterphasing at 2 Hz for 5 s per trial, with the first 10 trials unrecorded to allow the build-up of adaptation. Observers adapted to either a single Gabor in the target location, crowded-noise stimuli, or the flankers in isolation ('crowded-blank' stimuli). Two temporally separated intervals were then presented, one blank and the other with a single target Gabor oriented at either 45° (as with the adaptor) or 135° (Supplementary Fig. 3a). Each interval was presented for 200 ms and separated by 500 ms, with observers required to make a two-interval forced-

choice decision regarding which interval contained the target Gabor (regardless of orientation). The Michelson contrast of the test Gabors was under the control of two interleaved QUEST staircases, one for each test orientation, set to converge on 82% correct. Each run was repeated three times for each observer.

Threshold elevation ratios were determined by dividing post-adaptation thresholds by unadapted thresholds. A value of 1 thus indicates no effect, with higher scores indicating stronger levels of adaptation. Supplementary Fig. 3b depicts these values for each observer, with the mean effect in grey. Adapting to a single Gabor produced a three-fold elevation of detection thresholds for similarly oriented test Gabors (45°), with much less effect on test Gabors of the opposite orientation (135°). Adaptation to crowded-noise stimuli also induced a clear elevation of same-orientation thresholds, with around half the magnitude of the effect induced by a physically oriented target. Orientation selectivity was also evident with little, if any, threshold elevation for the detection of test stimuli oriented at 135°. Threshold elevation was smaller again for the crowded-blank stimuli, with elevation values only marginally above 1, though some orientation selectivity is nonetheless evident. In sum, both types of crowding stimuli induced an orientation-selective elevation of post-adaptation detection thresholds, despite the absence of a physically oriented stimulus in the target location. This is again consistent with the induction of flanker signals within the target representation, with the magnitude of threshold-elevation values for crowded-noise and crowded-blank stimuli further consistent with the different effect sizes seen with these stimuli in the change-detection paradigm (Fig. 2). It follows that our adaptation results cannot be explained in terms of the more general detection/identification dissociation that occurs under crowding [2, 3].

Previously reported effects of adaptation to crowded Gabors [9, 10], can be reconciled with our own results by our demonstration that crowding can produce an orientation-selective *change* in the target representation. This would have been obscured in prior studies because the effect of adaptation was examined only for similarly- and orthogonally-oriented test stimuli. Rather, were crowding to change the target orientation, the strongest adaptation may in fact be found at intermediate orientations due to observers adapting to a different orientation for a large proportion of trials. This would shift the tuning curve of adaptation effects away from the original target orientation, which would appear as a reduction in threshold elevation at the target orientation. Alternatively, it is possible that such change initiates local competition between the target and flanker-introduced signals, which could produce both changes in appearance and reduced adapting strength. These processes would be evident only when a target signal is present to interact with the flanker signals and would thus have been obscured with our stimuli.

Eye Movements and the Tilt Aftereffect

One issue with adapting to crowded stimuli is the requirement that flankers be close to the target in order for crowding to occur [4, 11]. Consequently, small eye-movements could cause the flankers to fall on the retinal area that would ordinarily be responding to the target stimulus, possibly contributing to adaptation at that locus. To test this possibility, we repeated the tilt aftereffect experiment (Fig. 3a) and made concurrent measurements of eye movements. Stimulus parameters were identical to those of the main experiment, though stimuli were presented on a Sony Trinitron monitor with a resolution of 1152×864 and a 100-Hz refresh rate. Eye movements were recorded using a head-mounted Eyelink I infrared gaze-tracker (SensoMotoric Instruments, Teltow, Germany) running at 250 Hz, in conjunction with the Eyelink toolbox for Matlab [12]. Calibration was conducted at the beginning of each

block of trials, with drift correction every five trials. Movements of the right eye were recorded during the 5.7 s period of each trial containing the adaptation stimulus (5 s), inter-stimulus interval (0.5 s) and test stimulus (0.2 s). Additionally, if fixation strayed more than 1.5 deg. on either side of fixation, trials were aborted and re-commenced. Recordings were not made during the response phase. All other procedures were identical to those of the main experiment. One observer (JAG) participated in this control study.

Results are depicted in Supplementary Fig. 4a (blue points) and show an identical pattern to those obtained previously (red points, data for JAG only). As before, orientations tilted slightly clockwise of the flanker orientation (45°) were seen as tilted further clockwise than prior to adaptation, and vice versa for counter-clockwise orientations, regardless of whether eye movements were recorded. Fixational jitter greater than 1.5 deg. caused trials to be aborted on less than 1% of the total. Supplementary Fig. 4b shows the concurrently measured distribution of eye-fixation positions, plotted in degrees of visual angle relative to the fixation point (grey triangle). Data have been normalised so that frequencies fall between 0-1, and smoothed using a Gaussian function with a standard deviation of 1 pixel. The majority of fixation positions cluster tightly around the fixation point, with X and Y standard deviations of 0.37 and 0.38deg, respectively. This is well within normal limits [13, 14].

Estimating the effect of this retinal smear requires some assumptions. If we take ±2 standard deviations around the fixation point, 95% of eye fixations were within ±0.75 deg. of the fixation marker. Given the spatial frequency and luminance contrast of our stimuli, we can then take their perceived size as ±2 standard deviations of the windowing Gaussian [15], to give a physical radius of 0.8 deg. Using these measurements, the impact of eye movements on our stimuli is shown schematically in Supplementary Fig. 4c. The target-noise patch is shown here with a rightwards flanker and a centre-to-centre separation of 2.75deg. Even when the estimated radius of our stimuli is added to the spatial extent of eye movements, the resultant shifts of the flankers would not exceed the separation between target and flanker stimuli. It is therefore unlikely that this fixational instability was sufficient to shift the flanker stimuli onto the region of the retina that would ordinarily be adapting solely to the target stimulus.

The Tilt Aftereffect and Uncrowded Stimuli

Although fixational eye movements were insufficient to move the flankers over the target location during adaptation, it is important that we consider the receptive field properties of the cells that would respond to these stimuli. In particular, it is well established that receptive fields grow in size as their location becomes increasingly peripheral [16], and as processing moves from V1 through to higher visual areas [17]. Given that our stimuli were presented at 15 deg. eccentricity, and that crowding may involve cortical regions beyond V1 [2, 18], it is likely that our stimuli activated cells with relatively large receptive fields. On the one hand, this is a potential model of the crowding process: the integrative operations typically seen in crowding tasks could result from a cortical resolution that is insufficient to represent the target and flanker stimuli independently [18, 19]. However, it could be that our stimuli simply adapt cells with large receptive fields in a manner unrelated to crowding. Given that our targets were either isotropic for orientation or absent altogether, the net effect could be adaptation to the flanker orientation (irrespective of whether crowding had occurred).

To test this hypothesis, we examined the effects of adaptation to a target stimulus surrounded by flankers that produce minimal crowding. Because crowding is tuned for orientation, flankers oriented orthogonally to the target produce little-to-no crowding, compared with

flankers oriented similarly to the target [20]. We thus compared the effect of adaptation to a single Gabor stimulus (oriented at 45°, as before) with that produced by adaptation to the same Gabor target surrounded by orthogonal flanking Gabors (oriented at 135°; Supplementary Fig. 5a). If the adaptation effects observed herein are related to the flanker orientation, regardless of crowding, the tilt aftereffect should either be reversed, due to the orthogonal orientation of the flankers, or reduced considerably in magnitude. On the other hand, if the strength of the adapting signal depends on the strength of crowding, the magnitude of the tilt aftereffect should be similar in both cases. To further minimize crowding in the orthogonal-flankers condition, all elements were presented at 100% peak contrast [21]. Accordingly, the orientation of the target Gabor was readily detectable under these conditions. The remaining parameters were identical to those of the adaptation experiment in-text (Fig. 3), and only observer JAG participated in this control experiment.

Results are shown in Supplementary Fig. 5b, where adaptation to a single Gabor in the target location again produces a robust tilt aftereffect, with a large repulsive effect that peaked at test orientations differing by ±10-15° from the adaptor. An identical pattern was produced following adaptation to the same Gabor target surrounded by four orthogonally oriented flankers, despite the potential for the orientation of these flankers to either produce the opposite direction of repulsion or reduce the magnitude of target adaptation. Flankers that produce minimal crowding thus have a minimal effect on the adapting strength of oriented target stimuli. It follows that our results are unlikely to have been produced by adaptation of cells with large receptive fields in a manner unrelated to crowding. Of course, it remains possible that crowding itself could be produced by integration across large receptive fields, as we consider in the General Discussion.

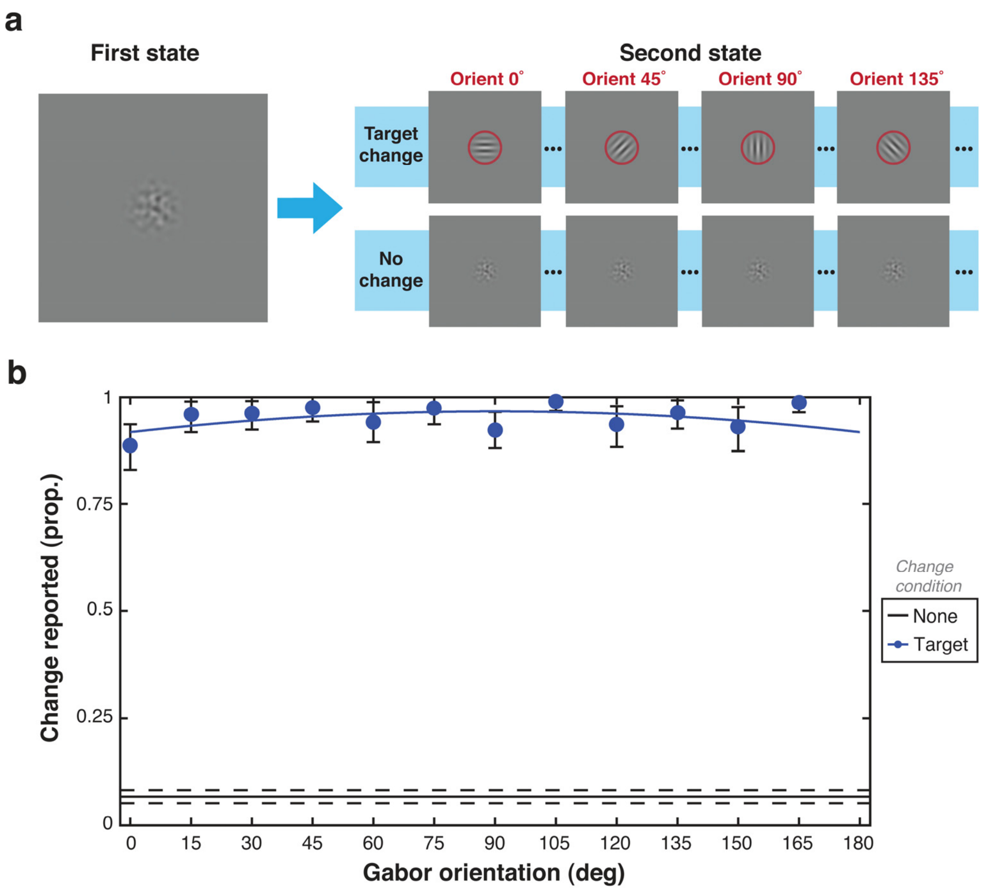

Figure S1. Uncrowded Change Detection

(A) Stimuli and procedure. Trials always began with target noise present, as in the main experiment. Here the noise was not crowded. Midway through each trial, the noise could either remain present (*no change*) or change into a Gabor (*target change*). All conditions were interleaved and observers indicated whether the target changed.

(B) The proportion of trials in which change was reported - pooled across three observers - is plotted separately for the *no change* and *target-change* conditions, the latter as a function of the absolute orientation introduced in the second stage. Error bars depict 95% confidence intervals. Performance is nearly identical across all orientations, indicating no difference in change-detection performance as a function of the introduced orientation.

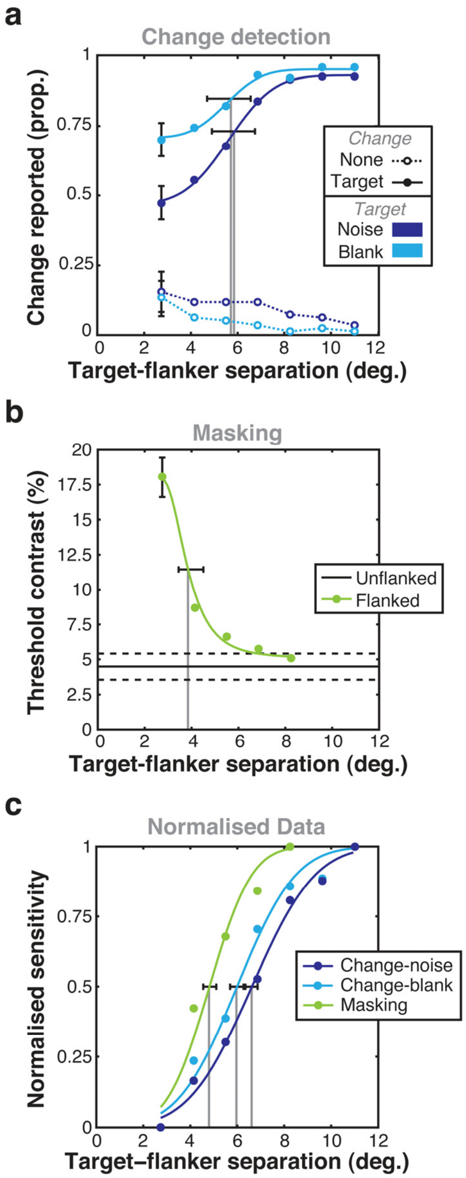

Figure S2. The Effects of Target-Flanker Separation

Data are pooled across three observers; error bars show 95% confidence intervals around the first data point of each series and the midpoints of each function, determined using bootstrapping.

(A) Change detection with crowded-noise (dark blue) and crowded-blank regions (light blue), plotted as the proportion of trials in which change was reported. Only the *no change* (dashed lines) and *target change* (solid lines) conditions were run, and introduced Gabors always matched the flankers. Change detection improves steadily with increasing target-flanker separation, with a half-width at half-height of 5.8 and 5.7 deg. for the crowded-noise and crowded-blank conditions, respectively (grey lines).

(B) Thresholds for detection of a Gabor target, with both unflanked (black line) and flanked (green points) configurations. Note that the x-axis shows the same target-flanker separations as panel A. Though there is some masking of target detection at the closest target-flanker separation, this rapidly declines to reach a half-width at half-height at 3.8 deg. separation (grey line), considerably less than that of crowded change detection.

(C) Normalized data for both masking and change-detection experiments. Sensitivity values were obtained by converting change-detection performance to d' and by inverting the threshold elevation ratios of the masking experiment. Data were normalized to between 0-1 by subtracting the minimum and dividing by the maximum. When fit with cumulative Gaussian functions, masking still drops off at a much faster rate than the two crowded-change conditions, with half-width at half-height values of 4.6, 6.0 and 6.6 deg., respectively.

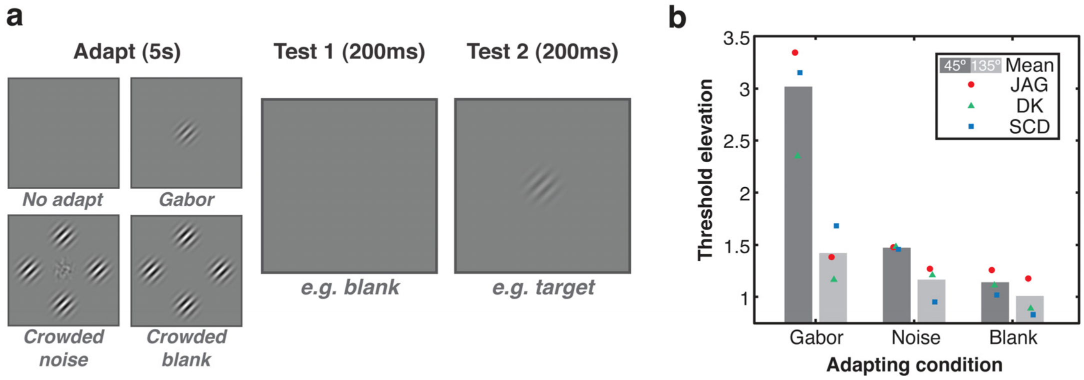

Figure S3. Adaptation and Detection
(A) Stimuli and procedure. As in the main experiment, observers were either (i) unadapted or adapted to (ii) a single Gabor in the target location (45° orientation), (iii) crowded noise stimuli with flankers oriented at 45°, or (iv) the flankers in isolation ('crowded blank'). Following 5-seconds of adaptation, two test intervals were sequentially presented for 200 ms each. One was blank and the other contained a single Gabor in the target location, oriented at either 45° or 135°. Observers indicated the interval containing the test Gabor.
(B) Threshold elevation values for the contrast-detection task. Results are shown for each observer, with mean values in grey. Adaptation to a single Gabor produced robust threshold elevation for detection of similarly oriented test stimuli (45°), with far less elevation for the opposite orientation (135°). Adaptation to crowded noise stimuli produced the same pattern at a smaller magnitude. Crowded-blank stimuli induced minimal adaptation, though some orientation selectivity is nonetheless evident.

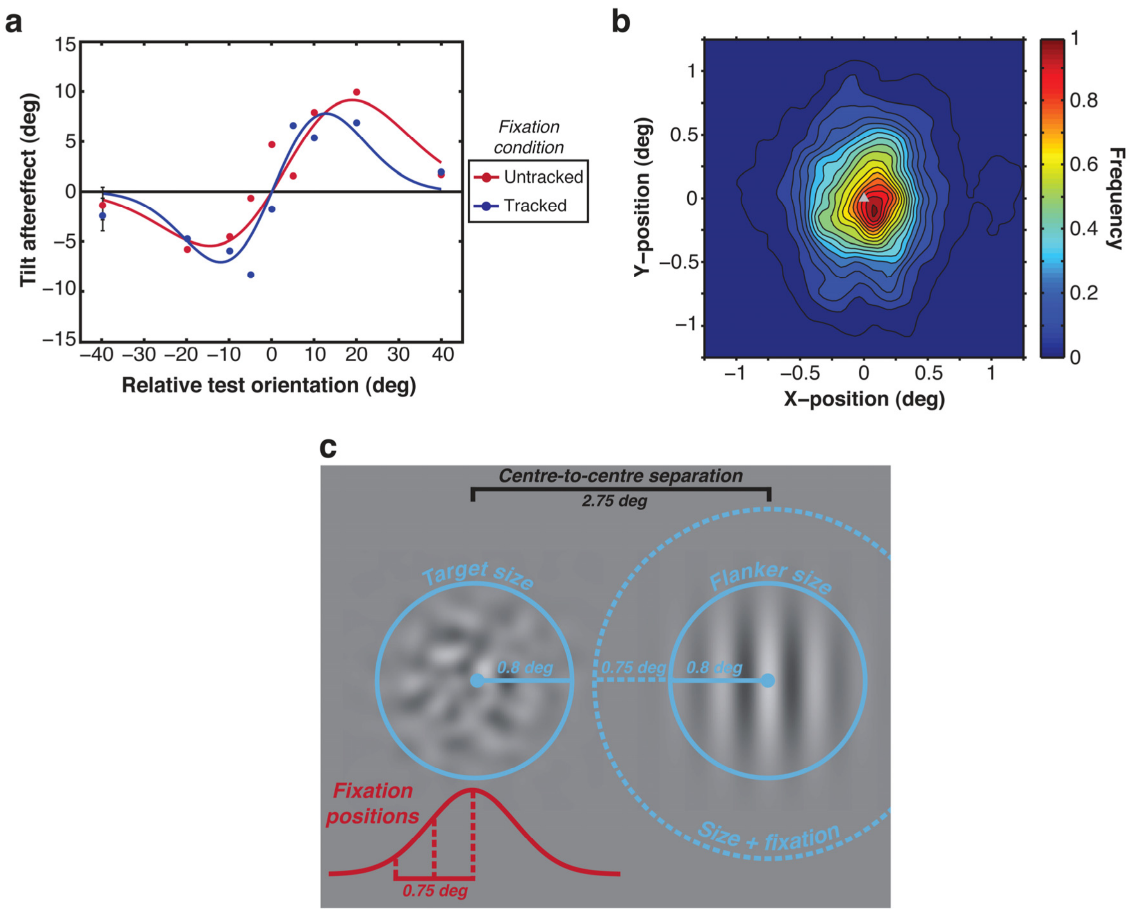

Figure S4. The Tilt Aftereffect and Eye Fixation

(A) The magnitude of the tilt aftereffect following adaptation to crowded-noise stimuli. Red points show the tilt aftereffect for JAG produced in the main experiment; blue points were measured with concurrent eye tracking. As before, negative values indicate clockwise rotations, positive indicate anti-clockwise rotations, and error bars depict ±1 SEM. In both cases, adaptation to crowded-noise stimuli produces a repulsion in perceived orientation that peaks at test orientations ±10-15° from the adaptor.

(B) The position of the right eye around fixation during adaptation and test intervals, relative to the fixation point indicated with a grey triangle at (0,0). Frequencies range between 0-1, and have been smoothed with a 1-pixel SD Gaussian function.

(C) A schematic view of the impact of eye movements on our stimuli. The radius of stimuli is estimated as 0.8 deg. (solid blue circles), given their spatial frequency and contrast, while the extent of eye movements is taken as ±2 standard deviations around the mean, giving a smear of 0.75 deg. in all directions (red distribution). The combination of these factors is depicted as a dashed blue line around the flanker, which does not exceed the centre-to-centre separation between flankers and the target.

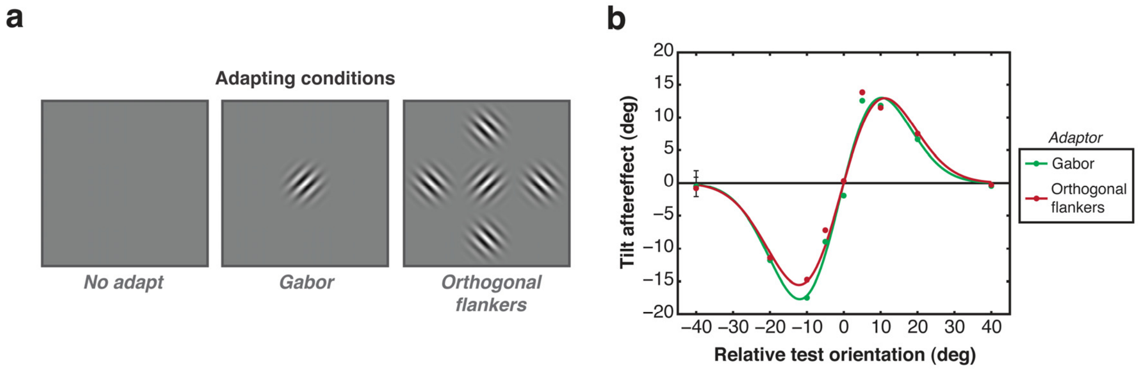

Figure S5. The Tilt Aftereffect and Uncrowded Stimuli

(A) Adapting stimuli: observers were either unadapted, or adapted to a single Gabor in the target location (45° orientation, 100% peak contrast) or a target Gabor at 45° surrounded by four flankers at 135° (‘orthogonal flankers’). The latter stimulus produces minimal crowding due to the dissimilarity between target and flanker stimuli.

(B) Changes in perceived orientation after adaptation, for observer JAG. Adaptation to a single high-contrast Gabor (green points) produced repulsion in the perceived orientation of test stimuli that peaks at orientations ±10-15° from the adaptor. An identical pattern is produced following adaptation to the orthogonal-flankers stimulus, despite the presence of the flankers.

Supplemental References

1. Efron, B., and Tibshirani, R.J. (1993). An introduction to the bootstrap, (London: Chapman & Hall).
2. Pelli, D.G., Palomares, M., and Majaj, N.J. (2004). Crowding is unlike ordinary masking: Distinguishing feature integration from detection. Journal of Vision *4*, 1136-1169.
3. Levi, D.M., Hariharan, S., and Klein, S.A. (2002). Suppressive and facilitatory spatial interactions in peripheral vision: Peripheral crowding is neither size invariant nor simple contrast masking. Journal of Vision *2*, 167-177.
4. Bouma, H. (1970). Interaction effects in parafoveal letter recognition. Nature *226*, 177-178.
5. Polat, U., and Sagi, D. (1994). The architecture of perceptual spatial interactions. Vision Research *34*, 73-78.
6. Chung, S.T.L., Legge, G.E., and Tjan, B.S. (2002). Spatial-frequency characteristics of letter identification in central and peripheral vision. Vision Research *42*, 2137-2152.
7. Watson, A.B., and Pelli, D.G. (1983). QUEST: A Bayesian adaptive psychometric method. Perception & Psychophysics *33*, 113-120.
8. Põder, E. (2008). Crowding with detection and coarse discrimination of simple visual features. Journal of Vision *8(4):24*, 1-6.
9. He, S., Cavanagh, P., and Intriligator, J. (1996). Attentional resolution and the locus of visual awareness. Nature *383*, 334-337.
10. Blake, R., Tadin, D., Sobel, K.V., Raissian, T.A., and Chong, S.C. (2006). Strength of early visual adaptation depends on visual awareness. Proceedings of the National Academy of Sciences of the United States of America *103*, 4783-4788.
11. Toet, A., and Levi, D.M. (1992). The two-dimensional shape of spatial interaction zones in the parafovea. Vision Research *32*, 1349-1357.
12. Cornelissen, F.W., Peters, E.M., and Palmer, J. (2002). The Eyelink Toolbox: Eye tracking with MATLAB and the Psychophysics Toolbox. Behavior Research Methods, Instruments & Computers *34*, 613-617.
13. Steinman, R.M. (1965). Effect of target size, luminance, and color on monocular fixation. Journal of the Optical Society of America *55*, 1158-1165.
14. Crossland, M.D., Morland, A.B., Feely, M.P., von dem Hagen, E., and Rubin, G.S. (2008). The Effect of Age and Fixation Instability on Retinotopic Mapping of Primary Visual Cortex. Investigative Ophthalmolmology & Visual Science *49*, 3734-3739.
15. Fredericksen, R.E., Bex, P.J., and Verstraten, F.A.J. (1997). How big is a Gabor patch, and why should we care? Journal of the Optical Society of America. A, Optics, Image Science, and Vision *14*, 1-12.
16. Hubel, D.H., and Wiesel, T.N. (1974). Uniformity of monkey striate cortex: A parallel relationship between field size, scatter, and magnification factor. The Journal of Comparative Neurology *158*, 295-305.
17. Smith, A.T., Singh, K.D., Williams, A.L., and Greenlee, M.W. (2001). Estimating Receptive Field Size from fMRI Data in Human Striate and Extrastriate Visual Cortex. Cereb. Cortex *11*, 1182-1190.
18. Levi, D.M. (2008). Crowding - An essential bottleneck for object recognition: A mini-review. Vision Research *48*, 635-654.
19. Pelli, D.G., and Tillman, K.A. (2008). The uncrowded window of object recognition. Nature Neuroscience *11*, 1129-1135.

20. Wilkinson, F., Wilson, H.R., and Ellemberg, D. (1997). Lateral interactions in peripherally viewed texture arrays. Journal of the Optical Society of America. A, Optics, Image Science, and Vision *14*, 2057-2068.
21. Kooi, F.L., Toet, A., Tripathy, S.P., and Levi, D.M. (1994). The effect of similarity and duration on spatial interaction in peripheral vision. Spatial Vision *8*, 255-279.
